# A New Full Pose Measurement Method for Robot Calibration

**DOI:** 10.3390/s130709132

**Published:** 2013-07-16

**Authors:** Hoai-Nhan Nguyen, Jian Zhou, Hee-Jun Kang

**Affiliations:** 1 Graduate School of Electrical Engineering, University of Ulsan, Mugeo 2-Dong, Namgu, Ulsan City 680-749, Korea; E-Mails: nhan.nguyenhoai@yahoo.com (H.-N.N.); freesoulzhou@hotmail.com (J.Z.); 2 School of Electrical Engineering, University of Ulsan, Mugeo 2-Dong, Namgu, Ulsan City 680-749, Korea

**Keywords:** full pose measurement, robotic manipulator, robot calibration

## Abstract

Identification of robot kinematic errors during the calibration process often requires accurate full pose measurements (position and orientation) of robot end-effectors in Cartesian space. This paper proposes a new method of full pose measurement of robot end-effectors for calibration. This method is based on an analysis of the features of a set of target points (placed on a rotating end-effector) on a circular trajectory. The accurate measurement is validated by computational simulation results from the Puma robot. Moreover, experimental calibration and validation results for the Hyundai HA-06 robot prove the effectiveness, correctness, and reliability of the proposed method. This method can be applied to robots that have entirely revolute joints or to robots for which only the last joint is revolute.

## 1. Introduction

Advanced applications such as off-line programming, robot-based measurement, and special robot-assisted surgery use highly accurate robotic manipulators. In order to satisfy the accuracy requirements of these applications, robots should undergo a calibration process, requiring practical full pose (position and orientation) measurements of robot end-effectors. The measurements are then used to identify robot kinematic parameters (unknown or approximately known). To acquire a full pose measurement of a robot end-effector (particularly the orientation one), previous researchers have used appropriate measurement devices that are expensive, relatively slow, and difficult to set up. Therefore, the aim of this work was to build a new method of full pose measurement that is accurate, easy to apply, and less time consuming.

Several authors have presented a non-contact full-pose measurement of robot end-effectors using visual systems [1-9] which utilize two cameras to capture 3D images [2,3,7], but the accuracy is low. In order to enhance identification speed and reliability, other researchers put markers, grids [4], or light tripe [5,6] on targets. Other systems involved cameras fixed on end-effectors to view targets more closely instead of zooming, which reduces a camera's field of view [8,9]. These visual systems still have limitations for accurate measurement of position and orientation of robot end-effectors.

Omodei *et al.* designed an optical device for full pose measurement with greater accuracy, and applied it in the calibration of a SCARA robot [10]; this device is specific and cannot be utilized for general robot types. Everett and Driels attached a special apparatus to the last robot link that has an arrangement of intermediate points, and end-effector full pose measurements are obtained based on these points [11,12]. Everett [11] utilized a single point sensor technique and an orientation fixture to collect the full pose (position and orientation) of robot end-effectors, while Driels used a coordinate measuring machine (CMM) to measure the center positions of five balls arranged on specially designed end-effectors [12]. Both the methods of Everett and those of Driels are reliable for robot calibration, but they have disadvantages due to the manufacturing costs of the special tools attached on the last robot link; the tools need to be pre-calibrated before use, making the measurement process slow, laborious, and time-consuming. Most recently, Nubiola *et al.* [13] proposed a full pose measurement method of robot end-effector using a single telescoping ball-bar and two planar fixtures, each fixture bears three magnetic cups which are located at the vertices of an equal triangle. This device has a hexapod geometry. This method [13] has some advantages such as highly accurate measurement, low cost and practical applications. However, it would be more perfect if one could enlarge the measurement range, reduce laborious involvement, eliminate the need for prior calibration of fixtures, and increase the number of measurable orientations.

**Figure 1.**
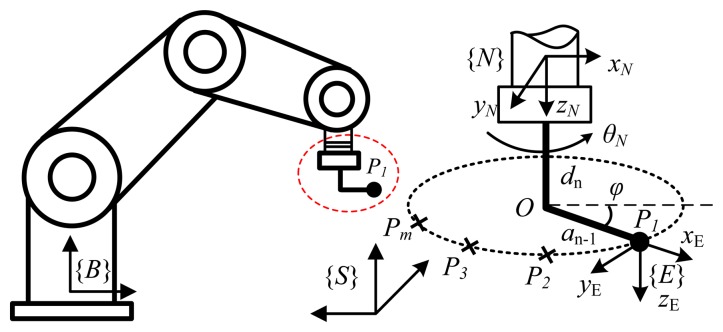
Basic principle of the measurement method.

This research proposes a new method for full pose measurement of end-effectors for robot calibration. This method provides a robot's full pose based on a set of discrete points on a circular trajectory, measured by a non-contact 3D coordinate measuring device (e.g., a laser tracker). Devices that utilize laser interferometry are widely used due to their high accuracy, fast measurement, large measuring range, and ease of use [14-17]. For robot configuration, the trajectory of a target fixed on a robot end-effector (tool) is measured when the end-effector is rotated uniquely; in this way one could obtain an arc of a circle and its center point *O* located on a rotation axis. An orthogonal vector of the rotation plane (which contains the arc of the circle) determines the *z* axis of the coordinate frame assigned to end-effector {*E*}. A vector connecting an initial point *O* with a terminal point *P*_1_ (Figure 1) determines the *x* axis of frame {*E*}, and axis *y* completes this orthogonal coordinate system. The proposed method benefits from the high accuracy of available 3D point measuring devices, and can be automated; therefore the application of the method is simple, relatively fast, and easy to set up. It also does not use any special tools that have additional manufacturing costs or need pre-calibration. The measurement accuracy of the method is evaluated by comparing deviation between two frames {*E*} and {*E*'} that are fixed on the robot end-effector, where frame {*E*} is computed by the proposed method, while frame {*E*'} is obtained by robot forward kinematics. The accuracy of the proposed method is evaluated via simulation on a Puma robot, and is demonstrated via experimental calibration on a Hyundai HA-06 robot.

In Section 2, the principles of the measurement method are presented and the plane and center points of rotation are identified. Section 3 presents an evaluation of the measurement accuracy of the method via a simulation on Puma robot, while Section 4 presents experimental calibration results for an HA-06 robot with full-pose measurements obtained by the proposed method. Section 5 presents our conclusions.

## 2. Principle of the Measurement Method

The proposed measurement method needs to determine two features: the rotation axis of the last robot joint and rotation center for the acquired position, and an orientation of the robot end-effector (*i.e.*, coordinate frame {*E*}) at every robot configuration. The basic principle of the measurement method is specified in three steps (Figure 1). In the first step, the measured trajectory of a target on a robot tool is a set of discrete points that lie on an arc of a circle when the last joint of the robot moves. In the second step, the rotation plane that contains the arc is identified. Finally, the center of the rotation situated on the last robot joint is also determined. The two features, which include the orthogonal vector of the rotation plane and the center of the rotation, determine the origin position and orientation of the coordinate frame {*E*} with respect to the sensor frame {*S*}. The two computation steps of the proposed method are identification of the plane and center point of rotation.

### 2.1. Identification of Rotation Plane

In order to establish a rotation plane, the last joint of an *N* degree of freedom (dof) robot is rotated while the other joints are locked. A target that is fixed on the robot end-effector generates an arc of a circle in Cartesian space; this arc contains the set of *m* positions of the target, which corresponds to the *m* angle positions of the last joint. A rotation plane that fits to this set of *m* points could be identified by applying a least squares algorithm.

A form of a rotation plane equation in Cartesian space can be presented as follows [18]:

(1) z=Ex+Fy+G,

where *x*, *y*, *z* are coordinates of points of the rotation plane, and *E*, *F*, *G* are coefficients of the rotation plane, which must be identified.

A plane, which fits a set of measured points (*x_i_*, *y_i_*, *z_i_*), *i* = 1,…, *m*, is obtained by solving the minimization problem for which the objective function is:

(2) $$J_{z}=\sum_{k=1}^{m}{\left(z-z_{k}\right)}^{2}.$$

The solution for Equation (2) in terms of the coefficients *E*, *F*, *G* can be found as follows:

(3) $$E=\frac{\sigma_{xx}\sigma_{yy}-\sigma_{zy}\sigma_{xy}}{\sigma_{xx}\sigma_{yy}-\sigma_{xy}^{2}},F=\frac{\sigma_{xx}\sigma_{zy}-\sigma_{xy}\sigma_{zx}}{\sigma_{xx}\sigma_{yy}-\sigma_{xy}^{2}},G=\bar{z}-E.\bar{x}-F.\bar{y}$$

the average values of coordinates in the set of *m* points on the arc of circle along directions *x*, *y*, *z* are computed as follows:

(4) $$\bar{x}=\frac{1}{m}\sum_{k=1}^{m}x_{k},\;\bar{y}=\frac{1}{m}\sum_{k=1}^{m}y_{k},\;\bar{z}=\frac{1}{m}\sum_{k=1}^{m}z_{k}$$

The covariance values in Equation (3) can be found as follows:

(5) $$\sigma_{xx}=\frac{1}{m}\sum_{k=1}^{m}{\left(x_{k}-\bar{x}\right)}^{2},\sigma_{yy}=\frac{1}{m}\times \sum_{k=1}^{m}{\left(y_{k}-\bar{y}\right)}^{2},\sigma_{xy}=\frac{1}{m}\sum_{k=1}^{m}\left(x_{k}-\bar{x})(y_{k}-\bar{y}\right)$$

(6) $$\sigma_{zx}=\frac{1}{m}\sum_{k=1}^{m}(z_{k}-\bar{z})(x_{k}-\bar{x}),\sigma_{zy}=\frac{1}{m}\sum_{k=1}^{m}\left(z_{k}-\bar{z})(y_{k}-\bar{y}\right)$$

where *m* is the number of measured target points corresponding to *m* positions of the last robot joint *N*.

**Figure 2.**
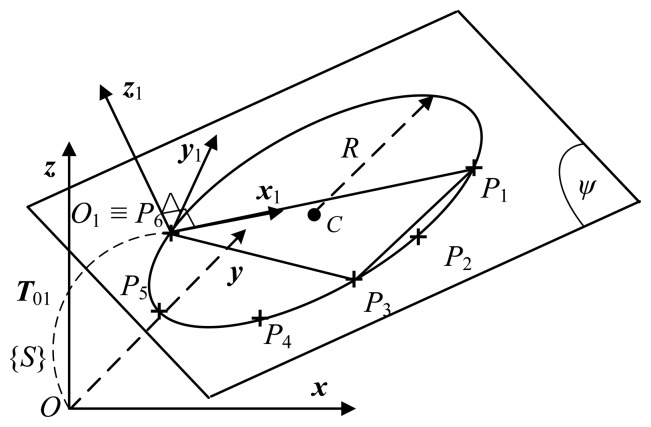
Point cloud transferring from frame *Oxyz* to frame *O*_1_*x*_1_*y*_1_*z*_1_.

The fitting plane is obtained by minimizing the objective function in Equation (2) in terms of the *z* coordinate of a sensor reference frame {*S*}. Therefore, in order to increase the accuracy of the fitting (or eliminate the systematic errors in the *z* axis), the measured points (point cloud) have to be transferred to another coordinate frame *O*_1_*x*_1_*y*_1_*z*_1_ such that the cloud of *m* points locates very closed to the plane *O*_1_*x*_1_*y*_1_ (the plane ψ in Figure 2) [19]. For simplicity, the frame *O*_1_*x*_1_*y*_1_*z*_1_ is going to be constructed for a case *m* = 6 including measured points *P*_1_, *P*_2_, *P*_3_, *P*_4_, *P*_5_ and *P*_6_ as in Figure 2. First, the farthest three points (*P*_1_, *P*_3_ and *P*_6_) among the six points are selected. A plane ψ, which contains the three points, is obtained. A normal vector of the plane determines the axis ***z***_1_. The axis ***x***_1_ is defined by unit vector $x_{1}=\vec{P_{6}P_{1}}/\left\|\vec{P_{6}P_{1}}\right\|$, then the axis ***y***_1_ completes the orthogonal coordinate frame *O*_1_*x*_1_*y*_1_*z*_1_ by the cross product of the known vectors: ***y***_1_ = ***z***_1_ × ***x***_1_.

Note that the frame *O*_1_*x*_1_*y*_1_*z*_1_ is arbitrarily located with respect to the reference frame *Oxyz* (sensor frame {*S*}). The points *P*_1_ – *P*_6_ are transferred to the frame *O*_1_*x*_1_*y*_1_*z*_1_. A plane fitting these points is obtained. Then, a circle that is on the identified plane and contains the points is also determined (see Subsection 2.2). The equations of the plane and the circle, which are currently expressed with respect to the frame *O*_1_*x*_1_*y*_1_*z*_1_, are going to be transferred back to the reference frame *Oxyz* for further using in the next step (see Subsection 2.3). As a result, an approximation of 3D point cloud using a plane and a circle will eliminate systematic errors in terms of the *z* axis of the reference frame.

### 2.2. Identification of Rotation Center

Theoretically, the trajectory of a point on a rotating rigid body about a fixed axis is a circle. This circle is on an orthogonal plane of the fixed axis. However, in practice, due to some factors (for examples assembly tolerance between spindle and bearing, vibration, measurement noise and so on) the trajectory can be a general curve (for example, an ellipse, circle and so on). If we assume that the deviation between the curve (ellipse) trajectory and the theoretical circle trajectory is sufficiently small, the deviation is then considered as noise. Therefore, a circle trajectory model will be used to fit these points.

After the above rotation plane is obtained, a least squares algorithm is applied to identify a circle that is on the identified rotation plane and contains the set of *m* measured points [18].

A standard form of a circle equation is as follows:

(7) $${\left(x-x_{c}\right)}^{2}+{\left(y-y_{c}\right)}^{2}=r^{2},$$

where (*x_c_*, *y_c_*) and *r* are the center and radius of the arc of the circle, respectively.

The circle Equation (7) can be rewritten in the following form:

(8) $$w=x^{2}+y^{2}=Ax+By+C,$$

The circle, which contains the set of measured points (*x*_i_, *y*_i_, *z*_i_), *i* = 1,…, *m*, can be obtained by solving the minimization problem for which the objective function is:

(9) $$J_{w}=\sum_{k=1}^{m}{\left(w-w_{k}\right)}^{2}.$$

A solution of Equation (9) in terms of the coefficients *A*, *B*, *C* can be computed as follows:

(10) $$A=\frac{\sigma_{wx}\sigma_{yy}-\sigma_{wy}\sigma xy}{\sigma_{xx}\sigma_{yy}-\sigma_{xy}^{2}},B=\frac{\sigma_{xx}\sigma_{wy}-\sigma_{xy}\sigma_{wx}}{\sigma_{xx}\sigma_{yy}-\sigma_{xy}^{2}},C=\bar{w}-A.\bar{x}-B.\bar{y}$$

where the average value *w̄* and co-variances in Equation (10) are computed as follows:

(11) $$\bar{w}=\frac{1}{m}\sum_{k=1}^{m}(x_{k}^{2}+y_{k}^{2}),$$

(12) $$\sigma_{wx}=\sum_{k=1}^{m}(x_{k}^{2}+y_{k}^{2}-\bar{w})(x_{k}-\bar{x}),\sigma_{wy}=\sum_{k=1}^{m}(x_{k}^{2}+y_{k}^{2}-\bar{w})(y_{k}-\bar{x}),$$

and the values of *x̄*, *ȳ*, *z̄*, σ*_xx_*, σ*_yy_*, σ*_xy_* are obtained from Equations (4, 5 and 6). *m* is the number of measured points of the target when rotating only the last robot joint *N*.

In this step the rotation plane is identified. The center point of rotation can also then be identified after some basic manipulation from Equation (8) to Equation (7). The two features of the measured point set are then available for the next derivation step of full-pose measurements.

### 2.3. Derivation of Full Pose Measurements

A system consists of a robotic manipulator with *N* dof and a 3D point sensing device as shown in Figure 1. The measurement process is executed in the following steps: first, at a specific robot configuration, a set *of m* Cartesian positions of target on end-effector is captured in terms of the sensor frame {*S*}. Next, a rotation plane and the center of an arc of a circle that fits these points are identified (see Subsections 2.1 and 2.2). Finally, a full pose measurement of the end-effector is derived by the following procedure:

‐ The robot configures itself such that a target on the end-effector comes to specific position *P*_1_.
‐ All robot joints are kept fixed except the last one, *N*. Next, the last joint is rotated in a positive direction such that the target comes to points *P*_2_,…, *P_m_* (*m* ≥ 3), and a set of these points are recorded.
‐ The set of points *P*_1_,…, *P_m_* are fitted with a plane in Cartesian space (see Section 2.1).
‐ On the determined plane, the arc of the circle that fits the set of points is identified (see Section 2.2), and its center *O* is also determined.
‐ An orthogonal vector of the rotation plane ***n***, which has unit length (║***n***║=1;, defines the axis ***z****_E_* of coordinate frame {*E*}. A vector that connects center point *O* and point *P*_1_, $v=\vec{OP_{1}}/\left\|\vec{OP_{1}}\right\|$ defines the axis ***x****_E_* of the frame {*E*}, Finally, the axis ***y****_E_* completes the orthogonal coordinate frame {*E*} by the cross product of the known vectors: ***y****_E_*= ***z****_E_* × ***x****_E_*.
‐ The full pose of the end-effector (*i.e.*, the pose of frame {*E*}) is described with respect to the sensor frame {*S*} as in the following transformation matrix:
(13) TSE=[xEyEzEp10001],
  where the relative position of frame {*E*} with respect to frame {*S*} is determined by a column vector of coordinates of point *P*_1_: ***p***_1_ = [*x*_P1_*y*_P1_*z*_P1_]*^T^*

Transformation from the last link frame {*N*} to the tool frame {*E*} (Figure 1) requires the following basic transformations [20]: rotation about axis *z* with an angle *φ*; translation along axis *x* with distance *a_N_*; and translation along axis *z* with distance *d_N_*_+ 1_ (refer to [20] for more details).

## 3. Evaluation for Measurement Accuracy

The measurements acquired by the proposed method for the robot calibration process should have an accuracy level. Therefore, it is necessary to evaluate the accuracy of measurements before applying them in the calibration process. This evaluation is performed by comparing deviation (error) between two coordinate frames {*E*} and {*E*'}, where frame {*E*} is computed by the proposed method, and frame {*E*'} is computed by the direct forward kinematics of the robotic manipulator. Criteria for comparison are: average position errors computed by Equation (14); standard deviations of these position errors computed by Equation (15); average Euler angle errors (order *x*, *y*, *z*) computed by Equation (16); and standard deviations of these Euler angle errors computed by Equation (17):

(14) $$\Delta \bar{x}=\frac{1}{g}\sum_{k=1}^{g}(x_{E}-x_{E'}),\;\Delta \bar{y}=\frac{1}{g}\sum_{k=1}^{g}\left(y_{E}-y_{E'}\right),\;\Delta \bar{z}=\frac{1}{g}\sum_{k=1}^{g}(z_{E}-z_{E'}),$$

(15) $$\sigma_{x}=\sqrt{\frac{1}{g}\sum_{k=1}^{g}{\left(x_{E}-x_{E'}-\Delta \bar{x}\right)}^{2}},\;\sigma_{y}=\sqrt{\frac{1}{g}\sum_{k=1}^{g}{\left(y_{E}-y_{E'}-\Delta \bar{y}\right)}^{2}},\sigma_{z}=\sqrt{\frac{1}{g}\sum_{k=1}^{g}{\left(z_{E}=z_{E'}-\Delta \bar{z}\right)}^{2}},$$

(16) $$\Delta \bar{\alpha }=\frac{1}{g}\sum_{k=1}^{g}\left(\alpha_{E}-\alpha_{E'}\right),\;\Delta \bar{\beta }=\frac{1}{g}\sum_{k=1}^{g}(\beta_{E}-\beta_{E'}),\;\Delta \bar{\gamma }=\frac{1}{g}\sum_{k=1}^{g}(\gamma_{E}-\gamma_{E'}),$$

(17) $$\sigma_{\alpha }=\sqrt{\frac{1}{g}\sum_{k=1}^{g}{\left(\alpha_{E}-\alpha_{E'}-\Delta \bar{\alpha }\right)}^{2}},\;\sigma_{\beta }=\sqrt{\frac{1}{g}\sum_{k=1}^{g}{\left(\beta_{E}-\beta_{E'}-\Delta \bar{\beta }\right)}^{2}},\;\sigma_{\gamma }=\sqrt{\frac{1}{g}\sum_{k=1}^{g}{\left(\gamma_{E}-\gamma_{E'}-\Delta \bar{\gamma }\right)}^{2}},$$

where (*x_E_*, *y_E_*, *z_E_*) and (*x_E'_*, *y_E'_*, *z_E'_*) are the relative coordinates of the origins of frames {*E*} and {*E*'} with respect to the sensor frame {*S*}, respectively. (*σ_E_*, *β_E_*, *γ_E_*) and (*σ_E'_*, *β_E'_*, *γ_E'_*) are Euler angles of frames {*E*} and {*E*'} with respect to the sensor frame {*S*}, respectively [20]. Δ*x̄*, Δ*ȳ*, Δ*z̄* are average position errors between the origins of frames {*E*} and {*E*'}. *σ_x_*, *σ_y_*, *σ_z_* are standard deviations of these position errors between frames {*E*} and {*E*'}. Δ*σ̄*, Δ*β̄*, Δ*γ̄* are average Euler angle errors of frames {*E*} and {*E* '}. *σ_σ_*, *σ_β_*, *σ_γ_* are standard deviations of these Euler angle errors between frames {*E*} and {*E*'}. *g* is the number of measured robot configurations.

**Figure 3.**
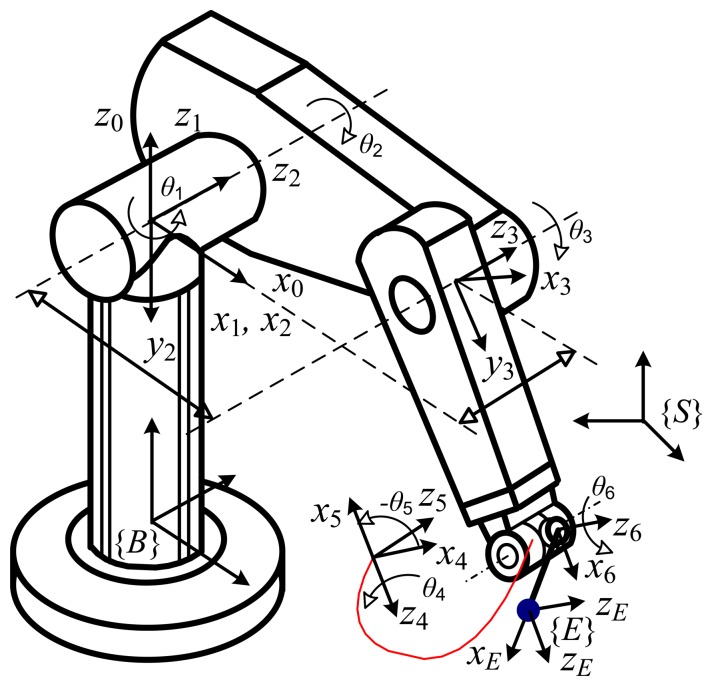
Robot PUMA and fixed link frames.

The accuracy of measurements acquired by this method can be evaluated via simulation for a specific robot. The typical Puma manipulator (Figure 3) was utilized in this evaluation process. The nominal model and corresponding Danevit Hartenberg (D-H) parameters of the Puma robot are presented in detail by Craig [20]. Applying the proposed method above, full pose measurements of robot Puma are obtained at a number of *g* = 30 robot configurations. The forward kinematic transformation determines the full pose of frame {*E*'} with respect to frame {*S*}. To compute the full pose of frame {*E*}, we rotate the last joint through *m* = 6 angle positions in positive angle increments of 20 degrees; a number of 6 target positions in Cartesian space can then be obtained, and full pose measurements of frame {*E*} with respect to frame {*S*} are then determined by the proposed method. Because measurement noise always exists in a practical measurement process and affects the accuracy of measurements, in this simulation Cartesian target positions should be corrupted by adding a (3 × 1) vector of random measurement noise values (Gauss distribution *N*[0,*σ*] with standard deviation σ = 0.01 [mm]).

Tables 1 and 2 present the computed comparison results. These results show that position and angle Euler errors are sufficiently small. Therefore, full pose measurements of the robot end-effector obtained by the proposed method are accurate enough for application in robot calibration.

## 4. Application of the Measurement Method in Practical Calibration

**Figure 4.**
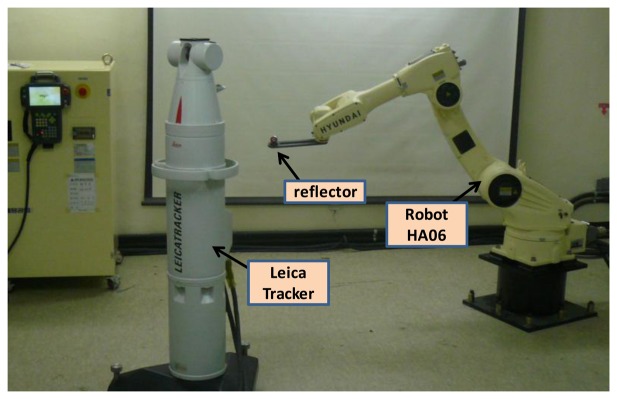
Experimental setup for calibration.

In order to identify robot kinematic errors we must first measure the position and orientation of the robot end-effector. This study applied the proposed measurement method in an experimental calibration for a Hyundai HA-06 robot. The system consisted of a Hyundai HA-06 robot (six dof and repeatability ±0.05 mm), a 3D measuring device (Laser Tracker LTD800 with accuracy of ±5 micron/m), and a laser reflector attached to the robot end-effector (Figure 4).

### 4.1. Kinematic Model of the HA-06 Robot

**Figure 5.**
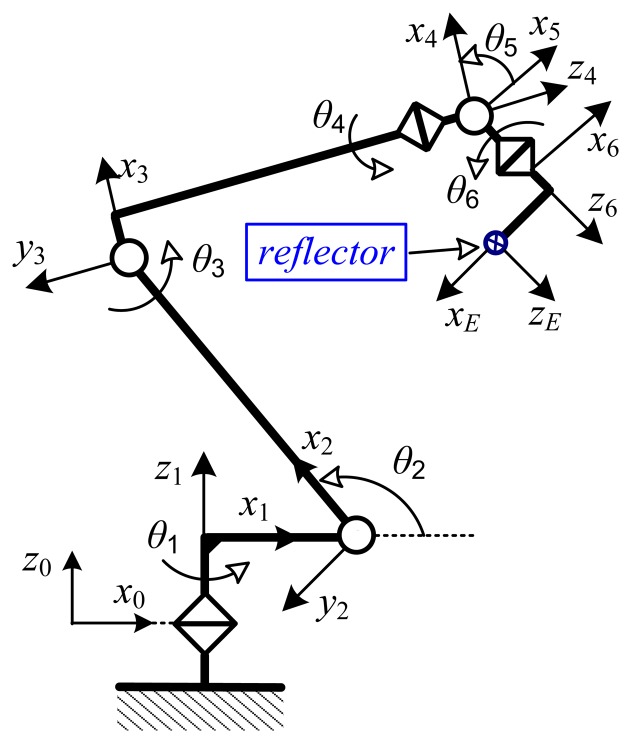
Schematic of robot HA-06 and attached link frames.

The nominal model of the HA-06 robot was established by using the D-H convention [21]. The frames are assigned from the robot base to the end-effector as in Figure 5. The according nominal D-H parameters are given in Table 3. A transformation from the base frame to the end-effector frame is computed as follows:

(18) TE0=T10T21T32T43T54T65TE6

where Tii−1 is a transformation matrix between two consecutive link frames {*i*-1} and {*i*}, *i* = 2 ÷ 6, and it is computed as follows:

(19) Tii−1=Rot(xi−1,αi−1).Tr(xi−1,ai−1).Tr(zi,di).Rot(zi,θi)

where link parameters are twist angles, link length, link offset and joint variables *α_i_*_−1_, *a_i_*_−1_, *d_i_* and *θ_i_*, respectively; *Rot*(·) and *Tr*(·) are (4 × 4) transformation matrices of purely rotation and translation about and along an axis, respectively [20].

The base transformation T10 can be computed from six basic transforms as follows:

(20) T11=Tr(x0,a0).Tr(y0,b0).Tr(z1,d1).Rot(x0,α0).Rot(y0,β0).Rot(z1,θ1)

where (*x*_0_, *y*_0_, *z*_1_) and (*α*_0_, *β*_0_, *θ*_1_) are translation and rotation parameters.

The parameters *y*_0_ and *β*_0_ do not exist in the nominal robot model, however, *y*_0_ and *β*_0_ must be included in the calibration robot model. The tool transformation TE6 (sees Figures 1 and 5) needs basic transforms: rotation about axis *z* with an angle *φ*, translation along axis *x* with distance *a*_6_, and translation along axis *z* with distance *d_7_* as follows:

(21) TE6=Rot(z,φ).Tr(x,a6).Tr(z,d7).

Because the robot model Equation (18) is used for calibration, this nominal model should be slightly modified for a case in which the link has two consecutively parallel axes [22]. Then, the individual transform in Equation (18) is modified to satisfy the properties of the model: complete, proportionality and continuous [23]. Specifically, the transformation T32 is modified as follows:

(22) T32=Rot(x2,α2).Tr(x2,a2).Rot(y2,β2).Rot(z3,θ3)

where *β*_2_ is parameter of the link twist about axis *y*_2_.

Robot kinematic error sources can be classified into two types: link geometric errors and non-geometric errors (such as link, joint deformation, joint backlash and so on). Because the HA-06 robot is a light weight manipulator, we assume that the robot has high link and joint stiffness, so the robot pose errors are only caused by link geometric errors. The number of identifiable kinematic parameters of the HA-06 robot is *n* = 27 (*d_2_* and *d_3_* are dependent, *θ*_6_ and *φ* are dependent).

### 4.2. Mathematic Formulation for Identification

Robot calibration aims to identify robot model parameters to describe the most correctly its kinematics, consequently the robot pose accuracy is enhanced. This section presents a formulation for the identification of the aforementioned kinematic parameters.

A mathematical formula for error identification is obtained by differentiating homogenous transformation TE0 of Equation (18). This transformation describes a relationship between the robot's kinematic errors and its end-effector pose errors as follows [24]:

(23) Δx=JΔp

where Δ***x*** is a (6 × 1) column vector of three differential position errors (Δ*x*, Δ*y*, Δ*z*) and three differential orientation errors (*δx*, *δy*, *δz*) of the robot end-effector. Δ***p*** is a (*n* × 1) column vector of kinematic errors (*n* = 27 is the number of identifiable robot kinematic parameters); particularly, Δ***p*** = [Δ*α*_0_…Δ*α*_5_ Δ*a*_0_…Δ*a*_5_ Δ*d*_1_*…*Δ*d*_6_ Δ*θ*_1_*…*Δ*θ*_6_]*^T^*. ***J*** is a (6 × *n*) Jacobian matrix that relates vectors Δ***x*** and Δ***p***; each column in matrix ***J*** corresponds to each error parameter in vector Δ***p***, and is computed by the following formulas [25] (*i* = 1 ÷ 6):

(24) $$J_{\alpha_{i-1}}=\left[\begin{array}{c} x_{i-1}\times p_{i-1}\\ x_{i-1} \end{array}\right],\;J_{a_{i-1}}=\left[\begin{array}{c} x_{i-1}\\ 0 \end{array}\right],\;J_{\beta_{i-1}}=\left[\begin{array}{c} y_{i-1}\times p_{i-1}\\ 0 \end{array}\right],\;J_{d_{i}}=\left[\begin{array}{c} z_{i}\\ 0 \end{array}\right],\;J_{\theta_{i}}=\left[\begin{array}{c} z_{i}\times p_{i}\\ z_{i} \end{array}\right]$$

***x****_i_*, ***y****_i_*, ***z****_i_* are (3 × 1) directional vectors of the link frame {*i*}, ***p****_i_* is a (3 × 1) vector of position of the end-point (origin of end-effector frame {*E*}) with respect to frame {*i*}, and **0** is a (3 × 1) zero vector.

### 4.3. Experimental Results for HA-06 Robot Calibration

Measurement for calibration is performed for 56 different robot configurations. For each configuration all robot joints are fixed except for the last joint, which rotates through *m* = 6 angle positions with positive angle increments of +20 [deg], and six positions of a target on an arc of a circle (*m* = 6) are recorded. Finally, the full pose (position and orientation) of the end-effector is derived by the proposed procedure (presented above in Section 2.3). As a result, a set Q_1_ of 56 full end-effector poses is obtained. By using all 56 full poses we can formulate an over-determined system of 6 × 56 = 336 differential equations based on Equation (23). The solution of this system of equations in the sense of least squares is the robot kinematic errors [26] as follows:

(25) $$\Delta p={\left(J^{T}J\right)}^{-1}J^{T}\Delta x$$

**Figure 6.**
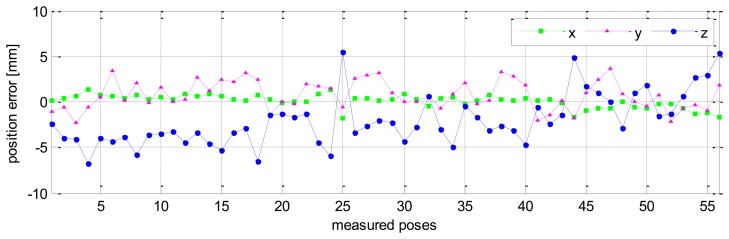
Robot position accuracy before calibration (pose set Q_1_).

**Figure 7.**
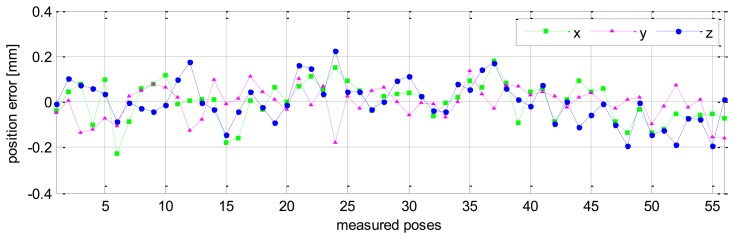
Robot position accuracy after calibration (pose set Q_1_).

**Figure 8.**
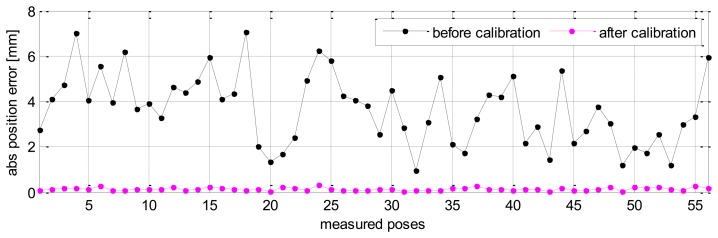
Robot's absolute position accuracy before and after calibration (pose set Q_1_).

**Figure 9.**
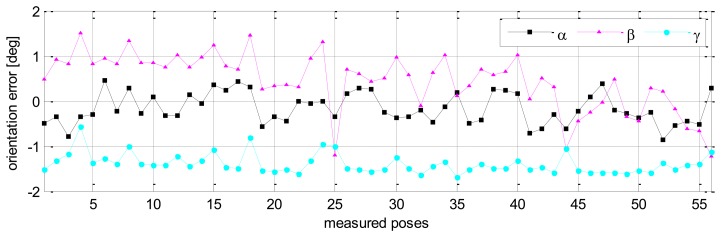
Robot orientation accuracy before calibration (pose set Q_1_).

**Figure 10.**
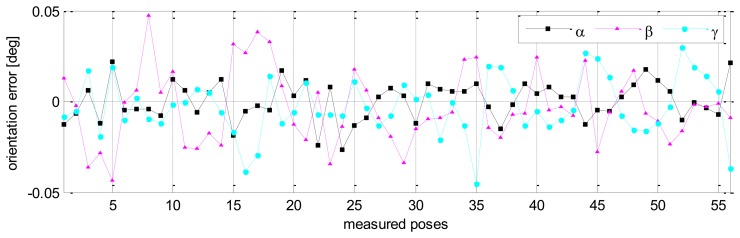
Robot orientation accuracy after calibration (pose set Q_1_).

Without calibration, the position accuracy of the HA-06 robot (which is computed over 56 measured configurations) is 3.6573 [mm], and its orientation accuracy about axes *x*, *y*, *z* (which is represented by Euler angle errors) is 0.33022, 0.67187, 1.4004 [deg], respectively (more details are shown in Table 4). After calibration, the position and orientation accuracy of the robot is significantly improved; particularly, the position accuracy is now 0.12933 [mm]; orientation accuracy is 0.00864 [deg] about axis *x*, 0.01658 [deg] about axis *y*, and 0.01286 [deg] about axis *z* (more details are also shown in Table 4). Figures 6 and 7 show the position accuracy of the robot for each configuration along directions *x*, *y*, *z* before and after calibration. The position accuracy along directions *x*, *y*, *z* is always in a range of [-0.3, +0.3] (mm). Figure 8 shows the absolute position accuracy at each measured pose before and after calibration. Figures 9 and 10 show the orientation accuracy of the robot for each configuration about axes *x*, *y*, *z* before and after calibration, respectively. The orientation accuracy about axes *x*, *y*, *z* is always in a range of [-0.05, +0.05] (deg).

The experimental results for the HA-06 robot show that robot pose accuracy is enhanced significantly after calibration. The proposed measurement method supplied the accurate full-pose measurements for this calibration and had some properties of effectiveness, correctness and practical applicability.

In many industrial applications, the robots have to operate over their full wide workspace. Therefore, a robot after calibration should have the same level of accuracy across its workspace. The pose accuracy of the HA-06 robot after calibration was validated with a set of 56 end-effector poses named Q_2_. The pose set Q_2_ was selected arbitrarily in whole robot workspace such that they were different from the set Q_1_. Note that the procedures for measuring both of the pose sets Q_1_ and Q_2_ are similar.

**Figure 11.**
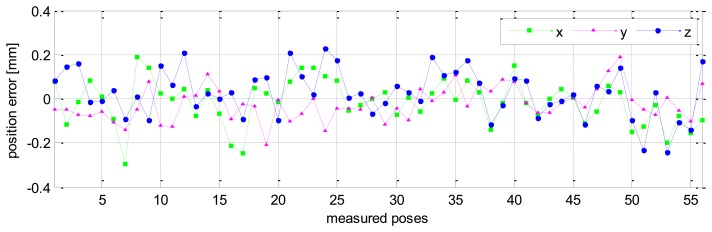
Robot position accuracy (validation, pose set Q_2_).

**Figure 12.**
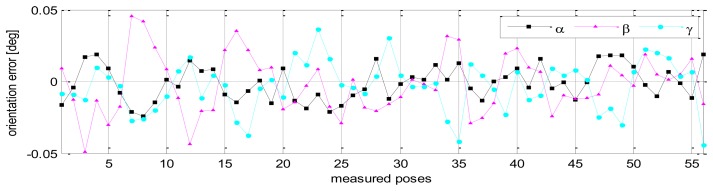
Robot orientation accuracy (validation, pose set Q_2_).

**Figure 13.**
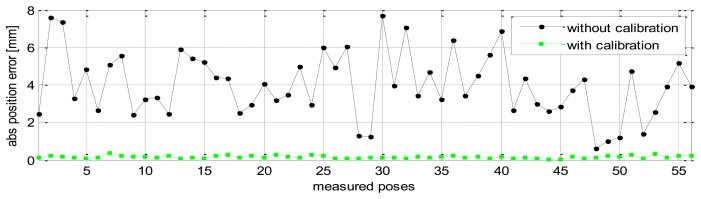
Robot's absolute position accuracy (validation, pose set Q_2_).

**Figure 14.**
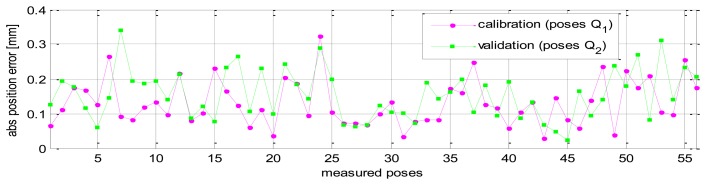
Absolute position accuracy at calibration poses Q_1_ and validation poses Q_2_.

The validation results of robot pose accuracy after calibration are illustrated in Figures 11, 12 and 13. Figure 11 shows the robot position accuracy along axes *x*, *y*, *z*. These values are in the range of [-0.3, +0.3] (mm). Figure 12 shows orientation accuracy about axes *x*, *y*, *z*. These values are in the range of [-0.05, +0.05] (deg). Figure 13 presents the robot's absolute position accuracy at the validation poses Q_2_ (without calibration: mean value is 3.989 [mm], maximum value is 7.7028 [mm]; with calibration: mean value is 0.1544 [mm], maximum value is 0.3403 [mm]). The absolute position accuracy with the pose set Q_1_ and the pose set Q_2_ are display on the same graph as in Figure 14. These figures show that the absolute position accuracy is less than 0.4 [mm]. The results in Figures 11, 12, 13 and 14 prove that the robot has the same level of pose accuracy over the workspace. After calibration, the absolute position and orientation accuracies robot HA-06 are better than 0.4 [mm] and 0.05 [deg], respectively.

## 5. Conclusions

This paper proposes a new method for the full pose measurement of an end-effector for robot kinematic calibration. Full pose measurements could be obtained by analyzing the features of a set of target points (designated on a robot end-effector) on an arc of a circle, such as an orthogonal vector of a rotation plane and a rotation center. These points are measured by using external point sensing devices. This method benefits from the accuracy of available point measurement devices, such as Laser Tracker. The measurement procedure is simple, fast, easy to set up, and can be automated. It also does not use any special apparatus with an arrangement of intermediate measured points; therefore, no additional manufacturing costs or pre-calibration steps are required.

The measurement accuracy of this method was gauged as sufficient based upon computational simulation results from the Puma robot. Experimental calibration for an HA-06 robot was performed to prove the practical effectiveness and accuracy of this method. The experimental results show that after calibration the HA-06 robot had enhanced position accuracy of 0.12933 [mm] (before calibration: 3.6573 [mm]), and increased orientation accuracy about axes *x*, *y*, and *z* of 0.00864 [deg], 0.01658 [deg], and 0.01286 [deg], respectively (before calibration: 0.33022 [deg], 0.67187 [deg] and 1.4004 [deg], respectively). These results provide evidence that the proposed method for full-pose measurement is accurate and reliable for practical robot calibration. The validation results also demonstrated that the HA-06 robot has the same level of pose accuracy over its full workspace. The new measurement method can be applied to robotic manipulators that have all joints or for which only the last joint is revolute. In the future we will evaluate the accuracy of this full pose-based calibration method on robots containing revolute and prismatic joints.

## Figures and Tables

**Table 1.** Origin position errors between frames {*E*} and {*E*'} for 30 robot configurations.

**Position Error**	Δ*x* [**mm**]	Δ*y* [**mm**]	Δ*z* [**mm**]
Average value	Δ*x̄* = 0.0024074	Δ*ȳ* = 0.0012843	Δ*z̄* = 0.0036070
Standard deviation	*σ_x_* = 0.0088443	*σ_y_* = 0.0117850	*σ_z_* = 0.0086101

**Table 2.** Euler angle errors between frames {*E*} and {*E*'} for 30 robot configurations.

**Angle Error**	Δ*α* [**deg**]	Δ*β* [**deg**]	Δ*γ* [**deg**]
Average value	Δ*ᾱ* = 0.0074211	Δ*β̄*= -3.315 × 10-^5^	Δ*γ̄* = 0.0063015
Standard deviation	*σ_α_* = 0.0319170	*σ_β_* = 0.005819	*σ_γ_* = 0.0345080

**Table 3.** D-H parameters of robot HA-06 (units: length [m], angle: [deg]; - : not exist, × : not select).

**i**	*α_i_*_-1_	*a_i-_*_1_	*β_i_* **_-_**_1_	*b_i_*_-1_	*d_i_*	*θ_i_*
1	0	0	0	0	0.36	θ_1_
2	90	0.200	-	-	0	θ_2_
3	0	0.560	0	-	0 (×)	θ_3_
4	90	0.130	-	-	0.620	θ_4_
5	−90	0.0	-	-	0.0	θ_5_
6	90	0.0	-	-	0.1	θ6 (×)
7	-	0.2	-	-	0.1	φ

**Table 4.** Absolute position and orientation accuracy of the robot over the set of 56 robot poses Q_1_.

		**Mean Value**	**Std.**	**Max. Value**
Absolute position accuracy [mm]	Before cal.	3.6573	1.55090	7.0433
	After cal.	0.12933	0.06618	0.32229
Absolute orientation accuracy about *x* axis: *α* Euler angle [deg]	Before cal.	0.33022	0.18441	0.86927
	After cal.	0.00864	0.00593	0.02649
Absolute orientation accuracy about *y* axis: *β* Euler angle [deg]	Before cal.	0.67187	0.36829	1.4892
	After cal.	0.01658	0.01164	0.0470
Absolute orientation accuracy about *z* axis: *γ* Euler angle [deg]	Before cal.	1.4004	0.22133	1.7003
	After cal.	0.01286	0.00962	0.0452
